# Erratum for Briese et al., “Virome Capture Sequencing Enables Sensitive Viral Diagnosis and Comprehensive Virome Analysis”

**DOI:** 10.1128/mBio.00615-17

**Published:** 2017-05-16

**Authors:** Thomas Briese, Amit Kapoor, Nischay Mishra, Komal Jain, Arvind Kumar, Omar J. Jabado, W. Ian Lipkin

**Affiliations:** a Center for Infection and Immunity, Mailman School of Public Health, Columbia University, New York, New York, USA; b Department of Epidemiology, Mailman School of Public Health, Columbia University, New York, New York, USA; c Department of Pathology and Cell Biology, College of Physicians and Surgeons, Columbia University, New York, New York, USA; d Department of Neurology, College of Physicians and Surgeons, Columbia University, New York, New York, USA

## ERRATUM

Volume 6, no. 5, e01491-15, 2015, https://doi.org/10.1128/mBio.01491-15. Influenza viruses A, B, and C were inadvertently labeled “no target” in Table S1 in the supplemental material. The revised table indicates that these viruses are targeted by the VirCapSeq-VERT probe set. The file for Table S1 has been replaced online.

